# Supplementary material for: Evidence accumulation in the pre-supplementary motor area and insula drives confidence and changes of mind
Source: Nat Commun. 2025 Jul 30;16:6998. doi: 10.1038/s41467-025-61744-8 (PMC12311133; doi:10.1038/s41467-025-61744-8)
Supplement: Supplementary file 1 — Supplementary Information [file 41467_2025_61744_MOESM1_ESM.pdf]

Supplementary information

Supplementary Notes

Model supplementary results

The fitted bound was 45.50 (SE = 3.59), the drift rate was 0.019 (SE = 0.002) and the non-decision time was 0.16 s (SE = 0.03). The bound for high confidence was 39.27 (SE = 3.12) and the readout time was 0.30 s (SE = 0.09) after the decision.

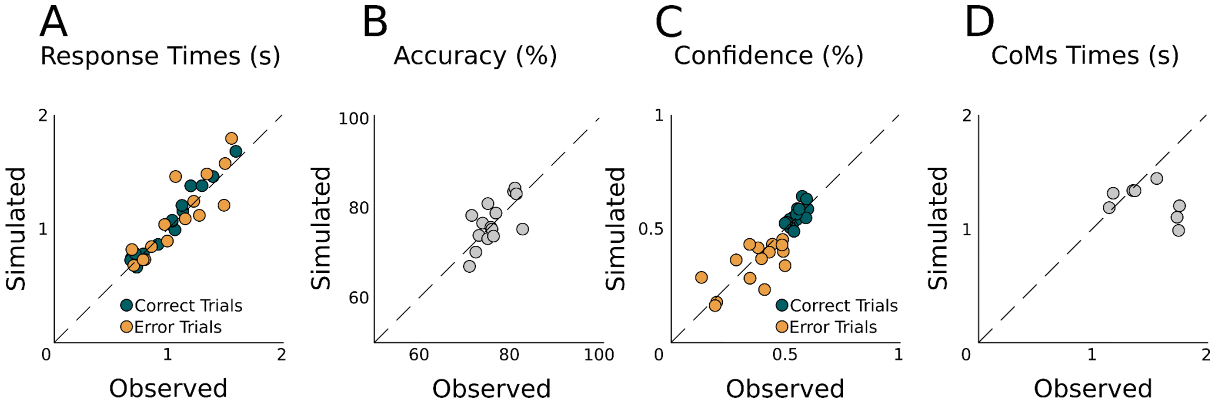

Figure S1. Between-participant correlation between observed and simulated data. A. Mean decision times (Spearman R = 0.96, p < 0.001 for correct trials, R = 0.90, p < 0.001 for error trials). B. mean response accuracies (R = 0.63, p = 0.014). C. Proportion of high confidence ratings (R = 0.96, p < 0.001 for correct trials, R = 0.90, p < 0.001 for error trials). D. Mean CoM timing (R = -0.24, p = 0.58)

Model fitting with control data

We also fitted our computational model to behavioural data from 13 healthy controls (7 females, mean age = 43.5, sd = 11.7). As observed with individuals with epilepsy, the model was successful in predicting performance, decision times and confidence.

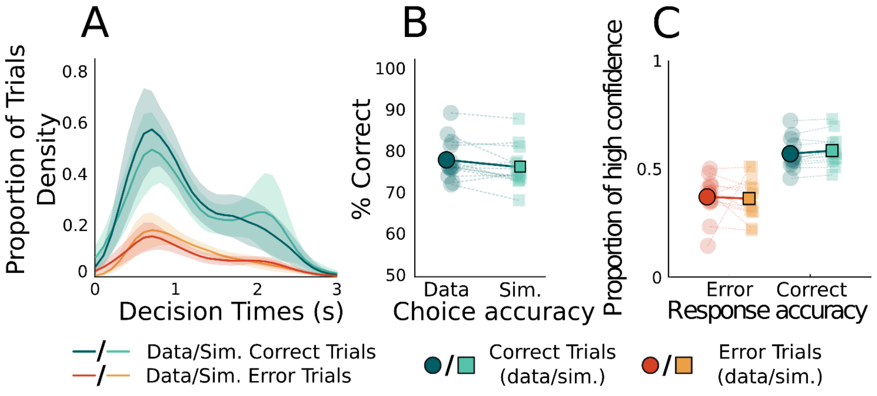

Figure S2. Model fits on behavioural data from healthy controls. Conventions are the same as in Figure 1. C. (**A**) Comparison of move onset timing density for data and model separated by correct and error trials (shaded areas represent 95% CI). **(B)** Choice accuracy from data and model prediction. Individual averages are plotted in light and population averages in darker green. **(C)** Proportions of trials with low/medium/high confidence for correct and error trials comparing data and model predictions. Same convention as the middle panel.

Regional correlates of confidence

The figure and analysis shown here are similar to Fig. 5 for the parietal and orbitofrontal cortices. We additionally display the regression coefficients, which were used for the latency analysis in Fig. 5C.

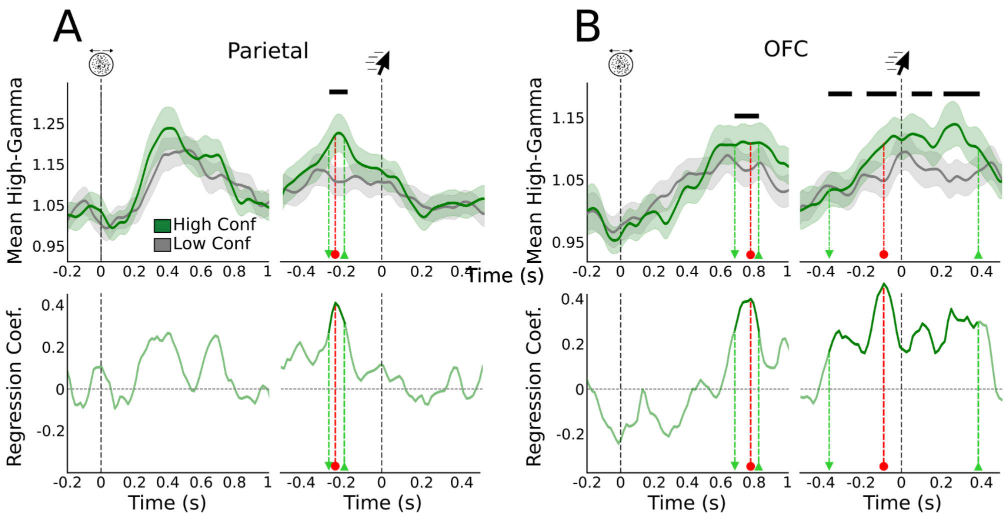

Figure S3. Stimulus-aligned and movement-aligned high-gamma activity for regions of interest reflecting evidence accumulation-like activity and confidence judgments (**A to B**) Top: Stimulus-aligned and movement-aligned high-gamma activity averaged across participants, including only EA-candidate channels in each ROI. Dark bars indicate a significant relationship between high-gamma and confidence ($p<0.05$, FDR corrected using the Benjamini-Hochberg method). Green lines correspond to averaged high-gamma for the 50% of trials with the highest confidence and grey lines for the 50% lowest. Shaded areas correspond to 95% CI. Bottom: Regression coefficient as a function of time in the stimulus and movement-aligned window. Light green markers indicate the onset and offset times of significant effects, and red markers indicate peak effects.

Performance and Confidence across nCoM and CoM trials

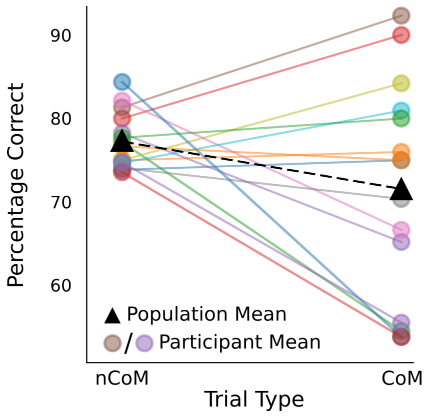

Figure S4. Comparison of average performance for non-change of mind and change of mind trial for each participant. Black triangles indicate population mean performance.

CoMs led to an overall decrease in accuracy (β = -0.306, p = 0.019), with high inter-individual variability. We also observed a significant negative effect of CoMs on confidence (β = -4.872, p<0.01). Such diversity in the effects of CoM on accuracy and confidence has already been reported in the same task Faivre et al., « Confidence in Visual Motion Discrimination Is Preserved in Individuals with Schizophrenia ».

Regional correlates of accuracy

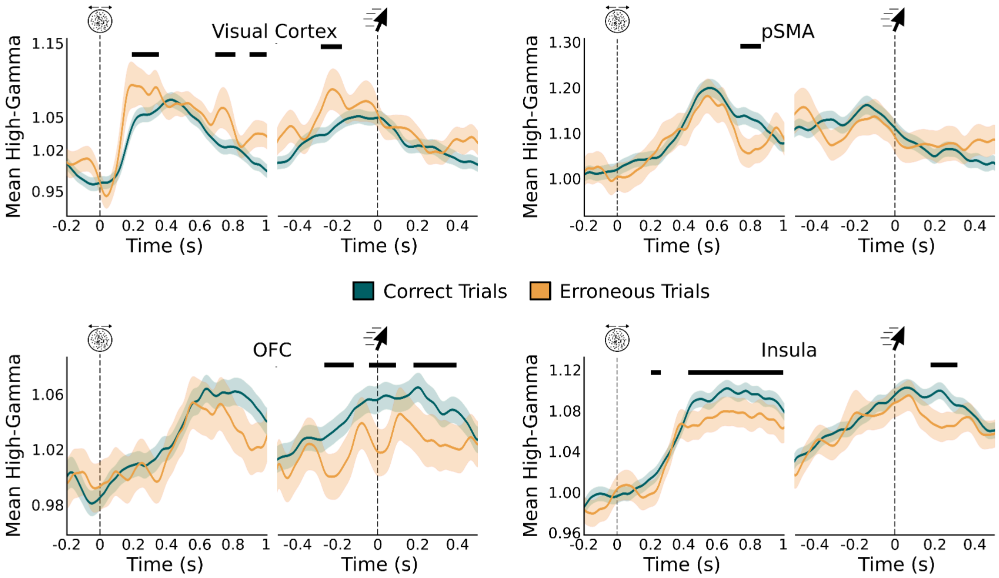

Figure S5. Stimulus-aligned and movement-aligned high-gamma activity averaged across participants for regions reflecting trial outcome, including all responsive channels. Dark bars indicate a significant relationship between high-gamma activity and trial outcome (p<0.05, FDR corrected using the Benjamini-Hochberg method). Dark blue lines correspond to averaged high-gamma for correct trials and light orange lines to erroneous trials. Shaded areas correspond to 95% CI.

Model simulations with different decisional/postdecisional evidence accumulation mechanisms

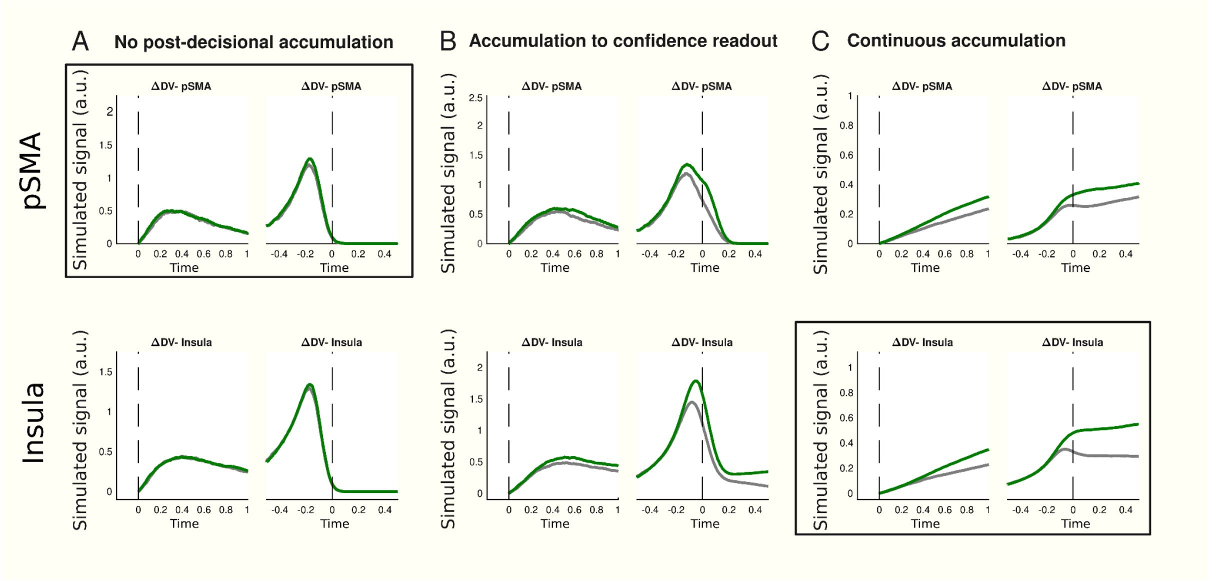

Figure S6. Comparison of model simulations for pSMA and Insula activity between **A.** accumulation-to-bound (accumulation stops after a decision boundary is crossed) and **B**. accumulation-to-confidence readout (accumulation continues for a few hundred milliseconds until confidence is read out) as in Figure 5A. **C.** Continuous accumulation (accumulation continues for an extended duration after a decision boundary is crossed). Left panels: time window locked on stimulus onset, right panels: time window locked on movement onset.

We selected single channels sensitive to accuracy by applying a generalised model for each channel for each time point following the decision time. Only 6 of the 102 responsive channels were found to be sensitive, and none exhibited a stronger activity following incorrect decisions. Although we did not find error sensitivity with single channel analysis, we performed a region-level analysis including all responsive channels to uncover if some ROIs encoded error signals. We performed a mixed generalised linear model analysis on the activity of all responsive channels pooled within each ROI. As shown in Fig. S5, four ROIs exhibited an effect of trial outcome on accuracy. However, none of the activity in these was systematically higher for correct trials, contrary to what would have been expected for error-monitoring signals.

Effect of sensory evidence on decision time

we ran a complementary analysis assessing the link between high gamma activity and response times with sensory evidence as a co-variate: HGA ~ decision time * sensory

evidence. This new analysis was performed at the level of regions of interest and revealed an effect of sensory evidence only for a short duration in the insula and no effect of interaction between decision times and sensory evidence (Fig. S7). The main effect of decision time was unchanged in all cases. We note that this analysis is not optimal as sensory evidence was titrated throughout the experiment using a 1up-2down staircase procedure, and therefore did not vary within participants to impact high gamma activity.

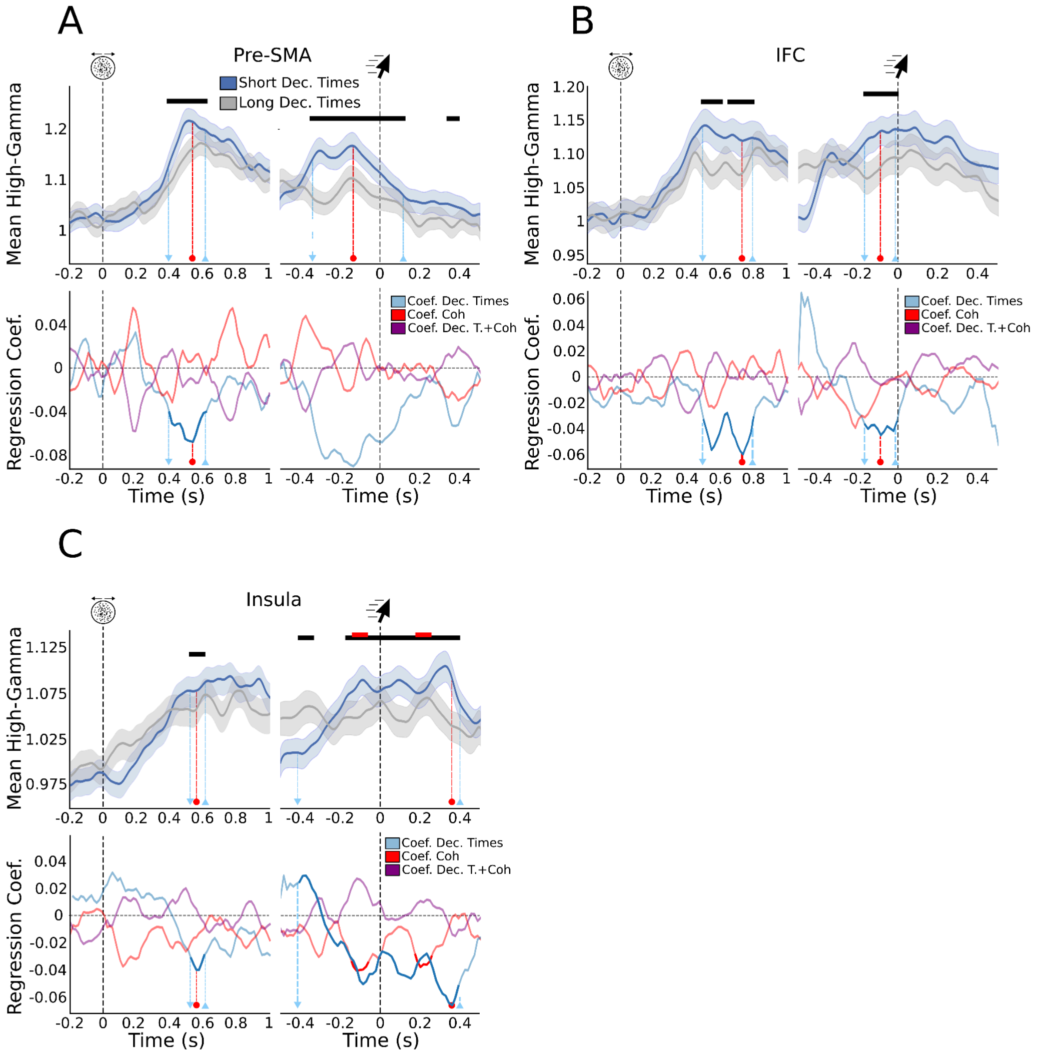

Figure S7. Stimulus-aligned and movement-aligned high-gamma activity for regions of interest reflecting evidence accumulation-like activity(**A, B, C**) Top: Stimulus-aligned and movement-aligned high-gamma activity (in arbitrary units) averaged across participants for all EA-candidate channels. Dark bars indicate a significant relationship between high-gamma activity and decision time, and red bars indicate a significant relationship between high-gamma activity and sensory evidence ($p<0.05$, FDR-corrected in both cases). Blue lines correspond to averaged high-gamma for the 50% fastest trials and grey lines for the 50% slowest. Shaded areas correspond to 95% CI. All analyses were performed on continuous decision times, and binarized only for illustrative purposes. Bottom: Regression coefficient as a function of time in the stimulus and movement-aligned window. Coefficients corresponding to the effect of decision time are shown in blue, the effect of sensory

evidence in red, and their interaction in purple. Light blue markers indicate the onset and offset times of significant effect of decision time, and the red marker indicates peak effects of decision time.

Additional analyses without exclusion criteria

To ensure that our exclusion criteria (excluding trials with erroneous responses or trials with overly delayed decision times) did not strongly impact our results we redid all of our analyses regarding the selection of evidence accumulation at the single channel level and the effects of decision time and confidence at the ROI level while including the previously excluded trials.

This manipulation left our results mostly unchanged, as illustrated in Fig. S8 for decision time and Fig. S9 for confidence.

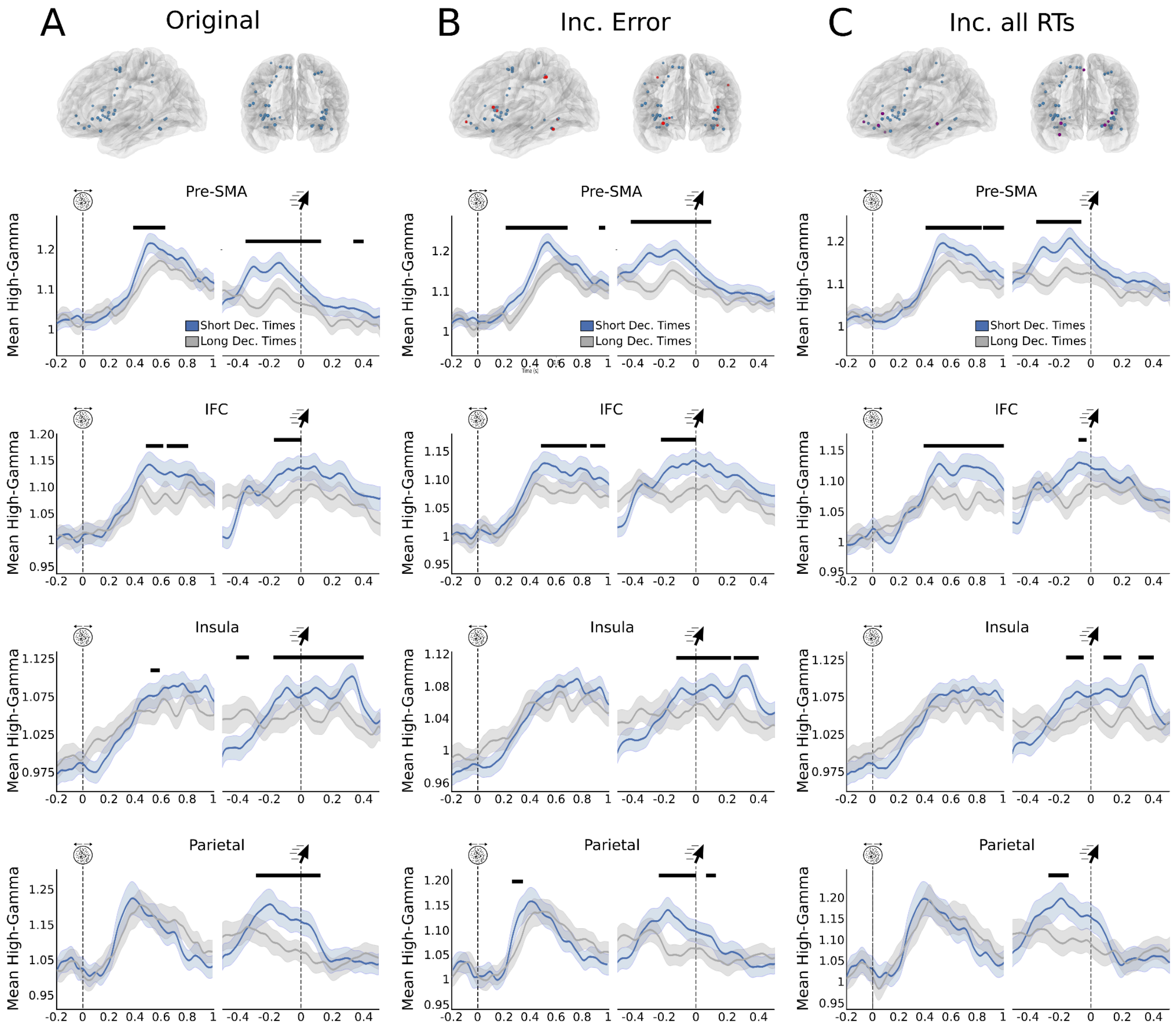

Figure S8. Results comparison between our original analysis (**A**), and similar analyses including error trials (**B**) and trials with decision times superior to 2.5s (**C**) Top (**A** to **C**): Template brain with channels reflecting EA-candidate channels. The original channels are shown in blue, the channels specific to the analysis including errors in red, and the channels specific to the analysis including all decision times in purple. Bottom (**A** to **C**) Same convention as in Fig.4. Each row corresponds to a region of interest. Dark bars indicate a significant

relationship between high-gamma activity and the decision time ($p<0.05$, FDR-corrected). Blue lines correspond to averaged high-gamma activity for the 50% fastest trials and grey lines for the 50% slowest. Shaded areas correspond to 95% CI.

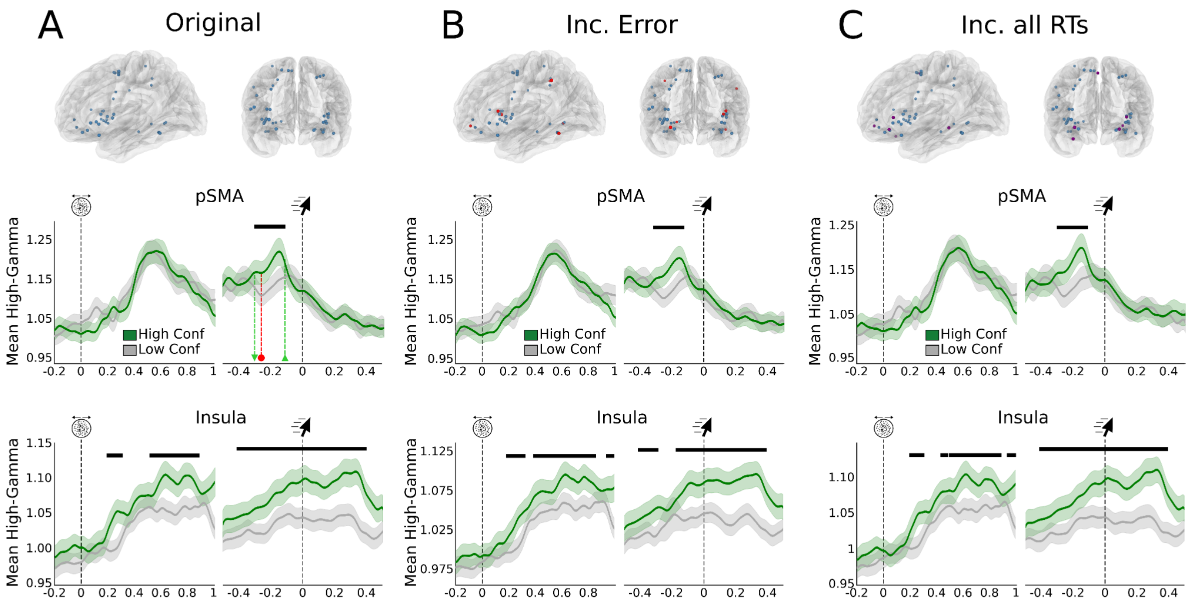

Figure S9. Results comparison between our original analysis (**A**), and similar analyses including error trials (**B**) and trials with decision times superior to 2.5s (**C**) Top (**A** to **C**): Template brain with channels reflecting evidence accumulation-like activity. The original channels are shown in blue, the channels specific to the analysis including errors in red, and the channels specific to the analysis including all decision times in purple. Bottom (**A** to **C**) Same convention as in Fig.5. Each row corresponds to a region of interest. Dark bars indicate a significant relationship between high-gamma activity and confidence ($p<0.05$, FDR-corrected). Green lines correspond to averaged high-gamma for the 50% trials with the highest confidence and grey lines for the 50% with the lowest confidence. Shaded areas correspond to 95% CI.

Computation of standardized coefficient for the effect of confidence in ROIs

In order to evaluate the comparative magnitude of the significant effect of confidence on high-gamma modulation observed in the pSMA and the Insula, we refitted a linear mixed-effects model, taking standardized high-gamma activity and confidence to obtain standardized coefficients. We obtained similar standardized beta coefficients for the pSMA and the Insula (0.188 and 0.193, respectively), indicating that the effect of confidence observed in these two ROIs is of similar magnitude.

Region of interest atlas

The selected ROIs were based on the Mars Atlas. The details regarding the seven ROIs as well as their corresponding Brodmann Areas (BAs) are provided below. Note that Mars Atlas-defined regions may cover only part of the associated Brodmann area.

ROI	MarsAtlas	Brodmann Areas
**Visual cortex**	- Caudal Medial Visual Cortex (VCcm)<br>Rostral Medial Visual Cortex (VCrm),<br>Cuneus (Cu)	BA 17/18<br>BA 18/19<br>BA 18/19
**Parietal cortex**	Superior Parietal Cortex (SPC)<br>Medial Superior Parietal Cortex (SPCm)<br>Dorsal Inferior Parietal Cortex (IPCd)	BA 7<br>BA 7<br>BA 39/40/7
**Pre-supplementary motor area**	Dorsolateral Premotor Cortex (PMdl)<br>Dorsomedial Premotor Cortex (PMdm)	BA 6/8<br>BA 6
**Dorsolateral Prefrontal Cortex**	Rostral Dorsolateral Inferior Prefrontal Cortex (Pfrdli)<br>Rostral Dorsolateral Superior Prefrontal Cortex (Pfrdls)	BA 10/46<br>BA 10/9
**Inferior Frontal cortex**	Rostral Ventrolateral Prefrontal Cortex (PFrvl)<br>Rostral Ventral Premotor Cortex (PMrv)	BA 10/9<br>BA 44/45
**OrbitoFrontal Cortex**	Ventral Orbito Frontal Cortex (OFCv)<br>Ventromedial Orbito Frontal Cortex (OFCvm)<br>Ventrolateral Orbito Frontal Cortex (OFCvl)	BA 11/47<br>BA 10/11<br>BA 11/47
**Insula**	Insular Cortex (Insula)	BA 13

Table S1. Region of interest definition

ROI	Resp. channels	EA channels	Conf. channels	Mixed channels	ROI level EA	ROI level EA (Vel. Model)	ROI level Conf.	ROI level Conf. (Vel. Model)	ROI level CoM
Visual Cortex	17 (N = 3)	8 (N = 2)	2 (N = 1)	2 (N = 1)	No	No	No	No	No
Parietal Cortex	8 (N = 3)	4 (N = 3)	3 (N = 1)	1 (N = 1)	No	No	Yes	Yes	No
pSMA	9 (N = 4)	7 (N = 3)	2 (N = 1)	2 (N = 1)	Yes	Yes	Yes	Yes	Yes
dlPFC	10 (N = 6)	1 (N = 1)	1 (N = 1)	0 (N = 0)	No	No	No	No	Yes
IFC	7 (N = 3)	6 (N = 4)	0 (N = 0)	0 (N = 0)	Yes	Yes	No	No	No
OFC	17 (N = 7)	4 (N = 4)	6 (N = 4)	3 (N = 3)	No	No	Yes	Yes	Yes
Insula	34 (N = 12)	11 (N = 5)	14 (N = 6)	7 (N = 3)	Yes	Yes	Yes	Yes	Yes
Total	102	41	28	15					

Table S2. channels repartition across ROIs and participants. Results at the ROI level are shown for simple models using decision time or confidence as a variable of interest and more complex models where instantaneous mouse velocity is added as a covariate.

Analysis of channels outside ROIs

To ensure that our ROIs selection did not skew our results we performed an exploratory analysis including all channels that were not included in the main article. We performed the same steps as described in the main text, namely selecting channels that were responsive to the stimulus, and among them identifying channels reflecting evidence accumulation and/or confidence. Of the total of 724 channels initially excluded from our analysis 69 were found to be responsive to the stimulus (9.53% of included channels, against 24.63% for the main text analysis). Among these, 31 channels were selected as EA-candidate channels, a number that significantly exceeded what was expected by chance (permutation test, $p = 0.0009$). Similarly, among the 69 responsive channels, 3 were found to reflect confidence, failing permutation testing (permutation test, $p > 0.01$). Considering that no significant cluster of channels exhibiting signatures of evidence accumulation or confidence emerged, we did not perform analyses at the level of ROIs.

Relationship between neural markers of confidence and decision time

Considering the correlation between decision times and confidence, we considered the possibility that the positive relationship we observed between high-gamma activity and confidence judgments may be partly driven by decision times. Although it is not possible to fully disambiguate the effects of decision times and confidence on the channels reflecting confidence, we could measure the respective strength of these effects and compare them. We did so by computing the correlation between high-gamma activity in a 400 ms window following the move onset and decision times and confidence. We reasoned that, if our effects were fully driven by decision times, the magnitude of the correlation with decision times should be stronger than with confidence. We found on the contrary that in this window the high-gamma levels correlated more strongly with confidence than decision times, confirming that the effect we observe is at the very least partly driven by confidence levels. The same results were found when considering the channels reflecting confidence in a 400ms pre-decisional window.

We also tested this potential confound at the population level by running a full linear mixed-effects regression, including confidence and decision time as independent variables as well as their interaction, on the clusters of interest in the pSMA and the insula with the following formula:

(Hga ~ Confidence*Decision Time + (1|Participant/channel))

The effect of confidence was preserved in all ROI, indicating that the interaction between confidence and decision time does not account for the effect of confidence on high-gamma activity.

Single participants model predictions

In this section we present the comparison between the model predictions and behavioural data for each participant included in the analysis.

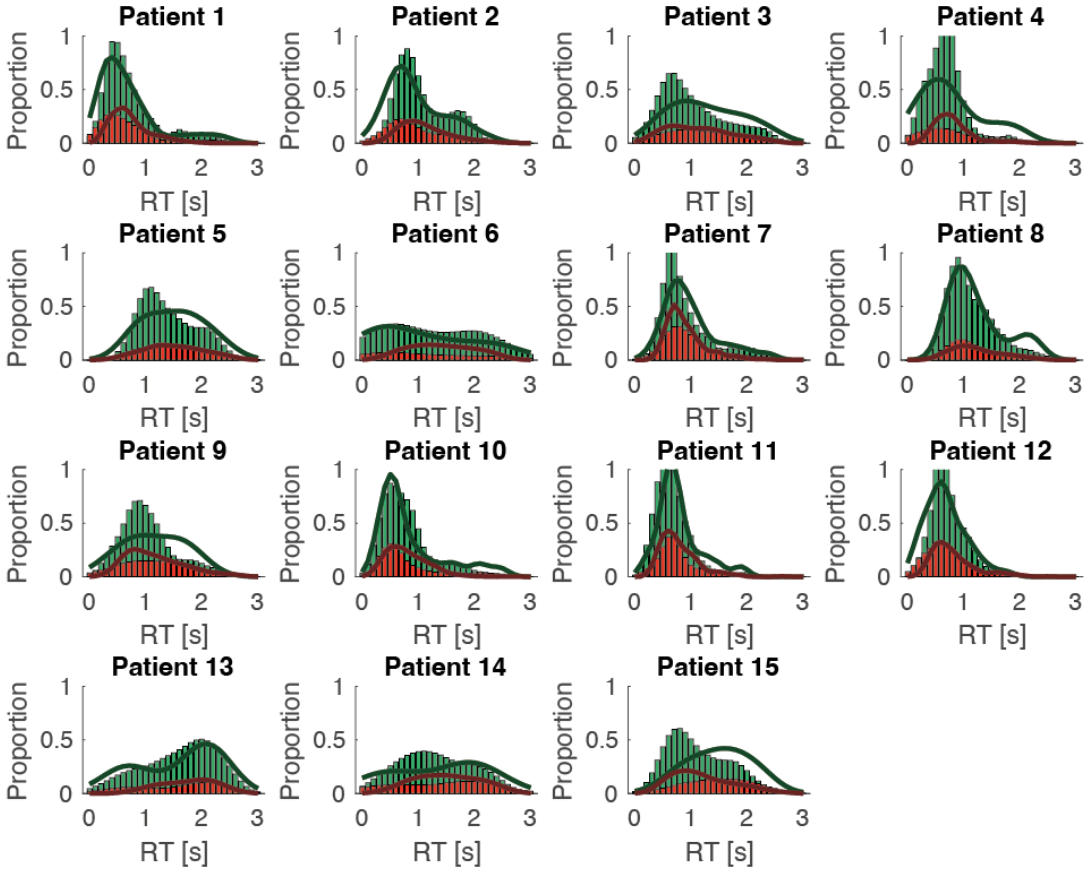

Figure S10.1 Individual proportion of response times for error (red) and correct (green) trials. Barplots show behavioral data and dark traces show model fits.

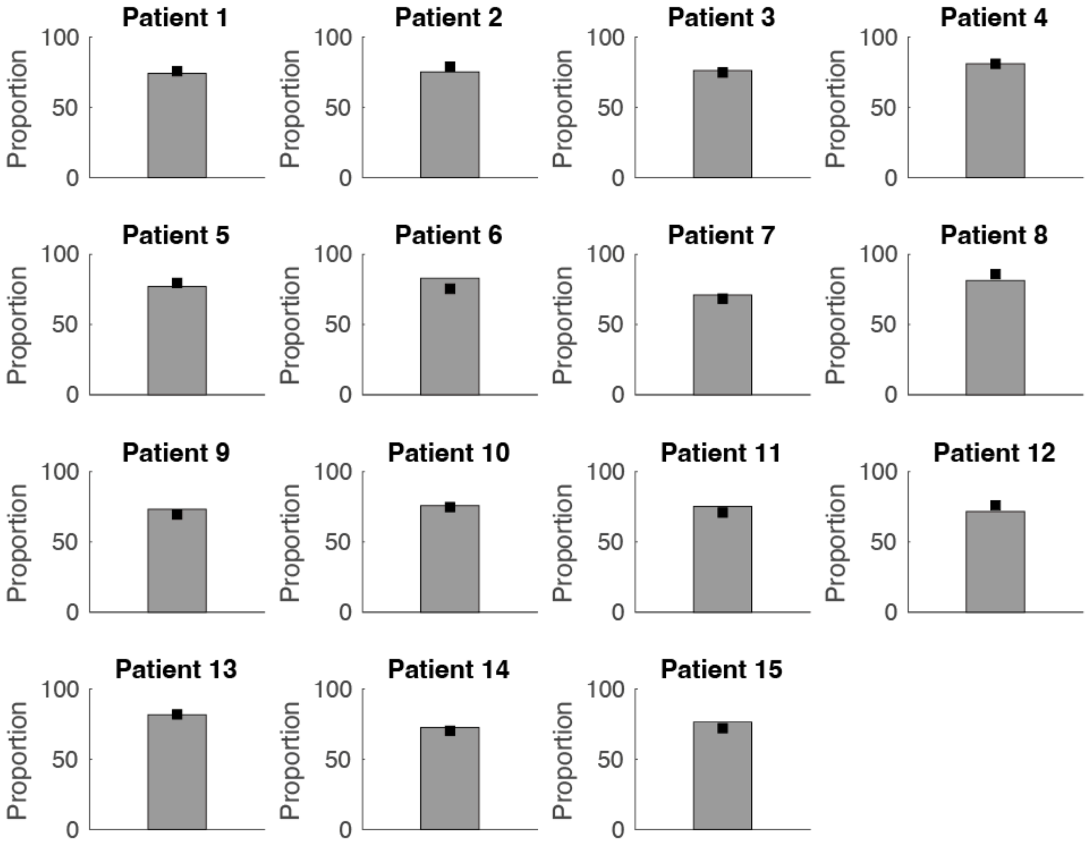

Figure S10.2 Individual proportion correct trials (correct direction of movement onset). Barplots show behavioral data and squares show model fits.

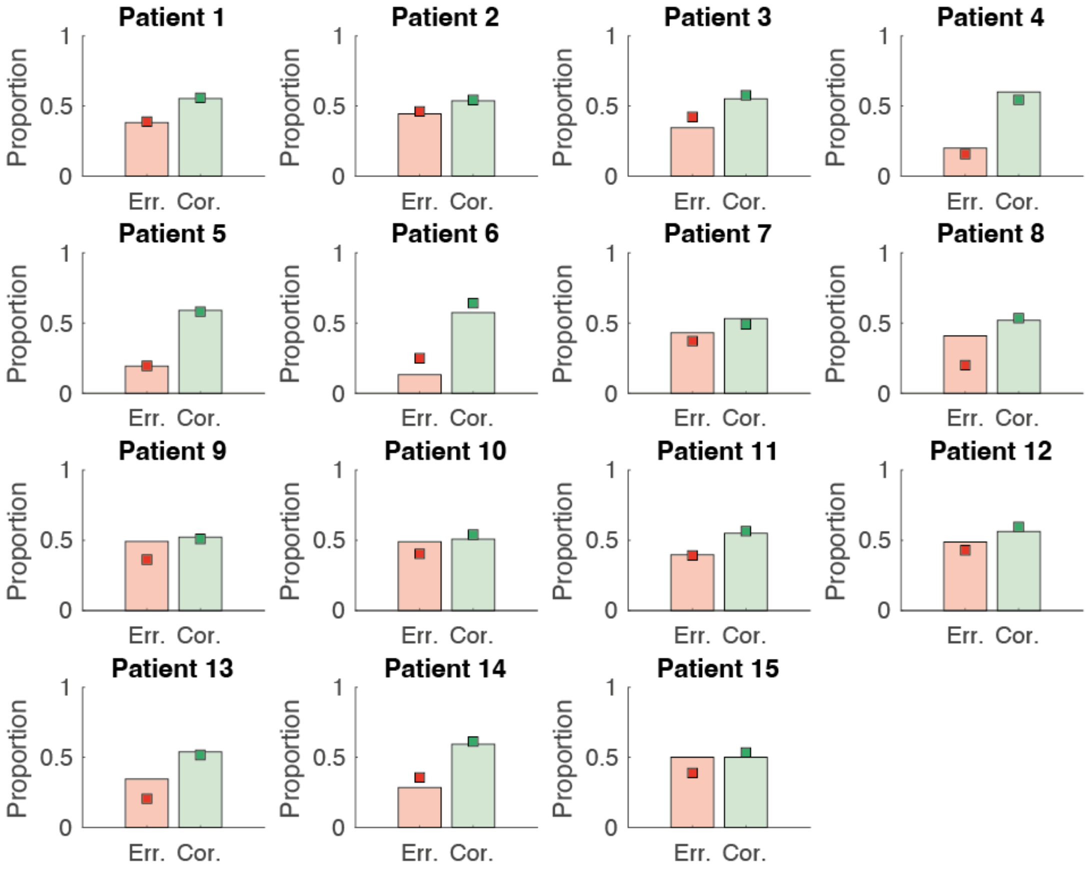

Figure. S10.3 Individual proportion of high confidence responses for error (Err.) and correct (Cor.) trials. Barplots show behavioral data and squares show model fits.

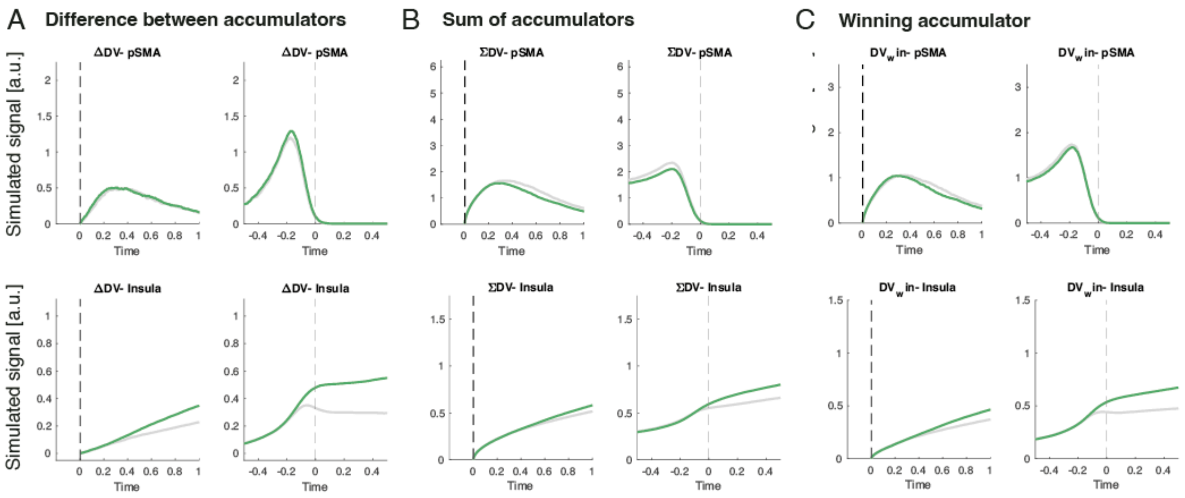

Figure S11. Neural predictions of simulated high-gamma activity for **A**. the difference between accumulators, **B**, the sum of accumulators and **C**, the winning accumulator.

Supplementary Discussion

Ruling out confounds regarding the markers of evidence accumulation

Our procedure for selecting EA-candidate channels could be confounded by a spurious relationship between the slope of high-gamma activity and decision time. This spurious relationship arises from the fact that we used the decision time to define the window during which the slope of high-gamma activity was calculated. Thus, this spurious relationship exists even if high-gamma activity rose to the same level with the same slope in every trial, independent of the decision time. We refer to this alternative hypothesis as the “stim-locked hypothesis”, distinct from the “EA-hypothesis” we argue for. We conducted several additional simulations and analyses to eliminate the possibility that this correlation could explain our results.

First, we simulated 10,000 response times resembling typical distributions, using a Gamma distribution plus 200 ms to avoid fitting slopes on too short windows (see the red trace on Figure S12). We then simulated 10,000 trials with a signal either linearly ramping until the response time (EA hypothesis), or ramping from 0 to 1 s then plateauing, independent of decision time, as expected under the stim-locked hypothesis. We added the same Gaussian noise to both simulations ($\mu = 0$, sigma = 0.1). Note that the results of these simulations were independent of the parameters (amount of noise or plateau time). We then computed the slope for each trial from stimulus onset to the decision time and correlated the resulting slope with decision time (as we did in the manuscript). We found correlations for both hypotheses, although at a much-reduced strength for the stim-locked hypothesis. We repeated this correlation 1,000 times while permuting the decision time values across trials. The resulting distribution corresponded to the correlations obtained in the stim-locked hypothesis, suggesting that any spurious or mechanical correlation would also occur in the permutations. This confirms that the permutation procedure we relied on to identify EA-candidate channels is efficient to compensate for this spurious correlation. We conclude from this analysis that our procedure might be too liberal in selecting high-gamma activity instantiating EA, but that our permutation procedure controls for that and therefore, our results cannot be observed under the stim-locked hypothesis.

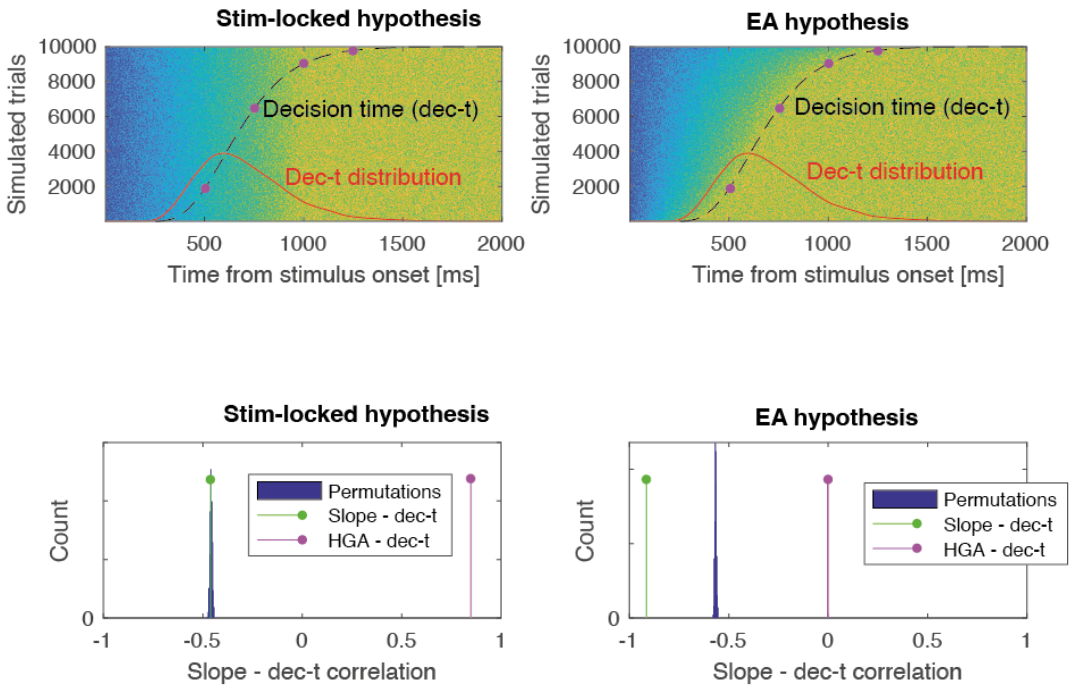

Figure S12. (Upper Panel) Heatmaps of simulated activity (sorted by decision time) for stim-locked (left) and EA (right) hypotheses. Dashed black lines represent the simulated decision time. The simulated decision time distribution over time is depicted in red.

(Lower Panel) Distribution of permuted high-gamma activity slopes vs. decision time (in blue) compared to the correlation obtained with simulated data (green dot). No difference between the correlation coefficient (green) and the coefficient distribution after permuting (blue) is expected under the stim-locked hypothesis (left panel). In other words, we expect to identify a similar number of channels with observed and permuted data and, therefore no statistical significance, which is not what we found. Under the EA hypothesis (right panel), the permuted correlation coefficients are expected to be smaller than the ones obtained with the simulated data, leading to a number of EA-selective channels that is higher than what would have been obtained by chance, as we report in the manuscript.

Confirmation of the soundness of the correlation with decision time over time.

The stim-locked hypothesis might explain the high-gamma slope vs. decision-time correlation found in *some channels*. However, if that were the case for all *channels*, first we would not find a significant number of channels with permutation tests, and second, we would not find effects of decision times on stimulus-locked high-gamma activity. We illustrated this latter point by repeating our analysis on the simulated data under both hypotheses (Fig. S13). This analysis confirms the absence of any difference under the stim-locked hypothesis, while we observe a steeper ramp-up for short decision times under

the EA hypothesis (Fig. S13), compatible with our observations at the ROI level (Fig.4).

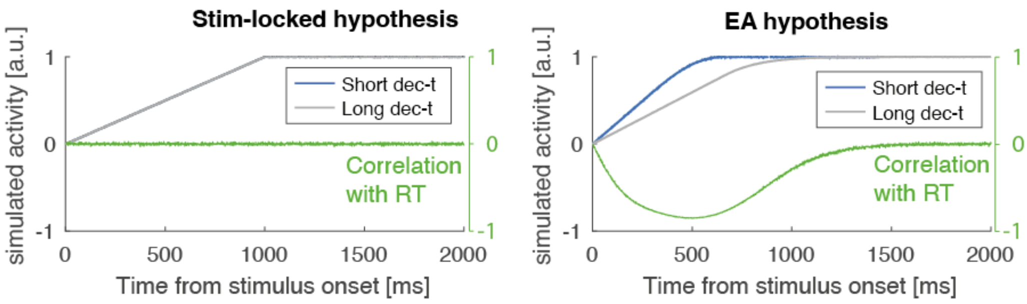

Figure S13. Simulated activity time-locked to the stimulus for short and long decision times (dec-t). Time-resolved correlations with decision times are shown in green, with a negative correlation for the EA hypothesis (right).

Finally, we examined the correlation between high-gamma activity at decision time and the decision time itself. The stim-locked hypothesis predicts a positive correlation between decision time and high-gamma activity. As shown in Fig. S14, none of the EA-candidate channels displayed such a correlation (the correlation between the mean high-gamma in a 100 ms window centered on decision time and decision time, Spearman Rank test, one-sided, all p-values > 0.05).

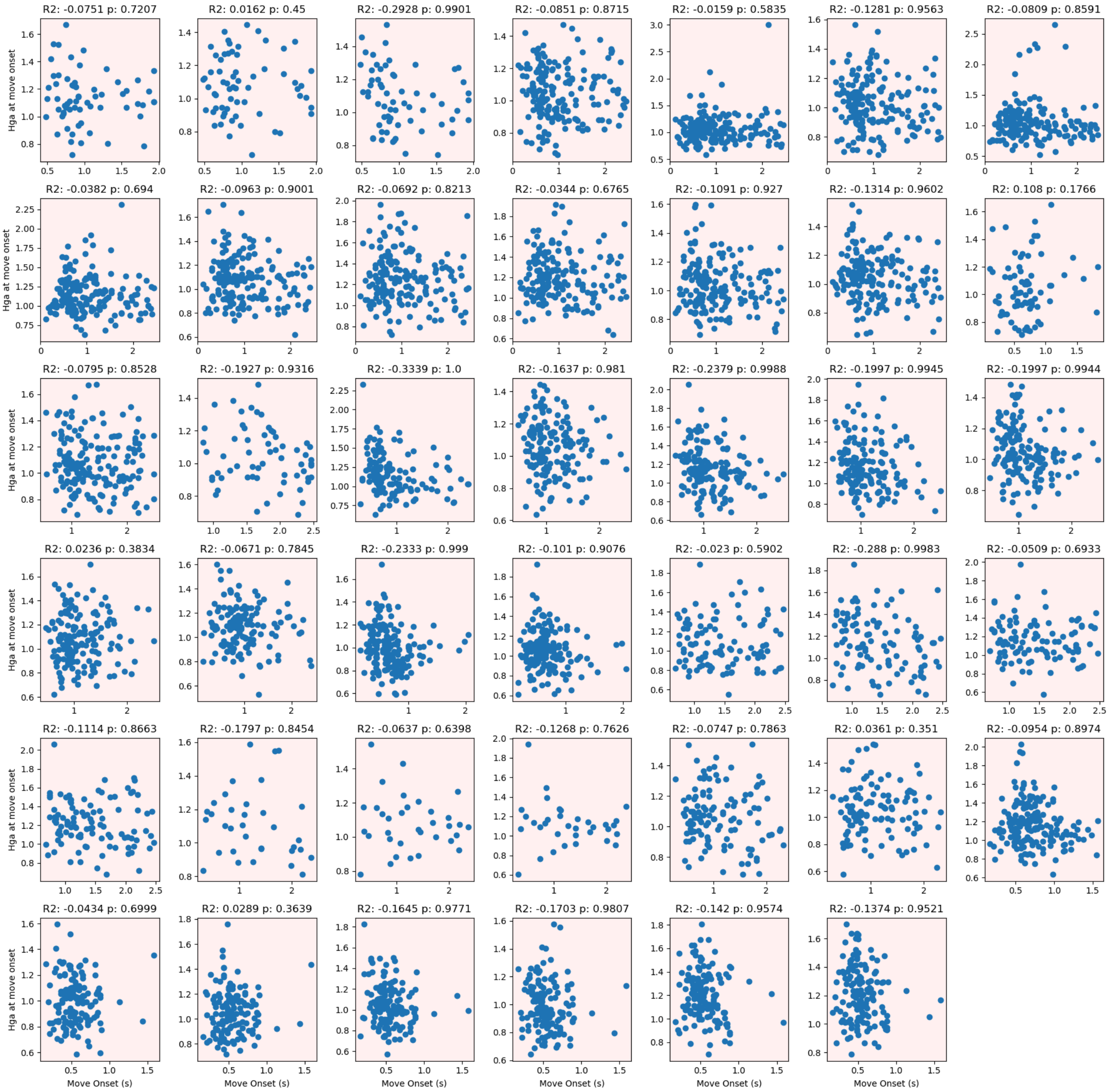

Figure S14. Correlation between decision time and high-gamma activity at decision time. Each subplot corresponds to the correlation between high-gamma activity at decision time and decision time for a particular EA-candidate channel. Red background indicates the absence of a significant positive correlation.

sub	Total channels	Removed	Remaining	Proportion remaining (%)
**1**	122	3	119	97.54
**2**	122	0	122	100
**3**	122	14	108	88.52
**4**	122	53	69	56.55
**5**	122	19	103	84.42
**6**	122	20	102	83.60
**7**	122	59	63	51.63
**8**	122	4	118	96.72
**9**	122	0	122	100
**10**	122	6	116	95.08
**12**	122	15	107	87.70
**13**	122	11	111	90.9
**14**	122	8	114	93.44
**16**	184	29	155	84.23
**17**	143	28	115	80.41
**18**	136	58	78	57.35
**19**	169	7	162	95.85
**20**	204	104	100	49.01
**21**	186	32	154	82.79
**22**	185	25	160	86.48
**23**	122	1	121	99.18
Total	2915	496	2419	
Mean	138.80	23.61	115.19	83.88
Std	27.87	25.98	26.80	16.24

Table S3. Fraction of channels removed during preprocessing

Participant	Age Group	Sex
**1**	39-48	M
**2**	18-28	F
**3**	18-28	F
**4**	39-48	F
**5**	39-48	M
**6**	18-28	M
**7**	18-28	F
**8**	29-38	F
**9**	18-28	M
**10**	49-58	M
**12**	18-28	M
**13**	29-38	F
**14**	18-28	M
**16**	18-28	M
**17**	39-48	M
**18**	18-28	F
**19**	29-38	M
**20**	18-28	M
**21**	39-48	F
**22**	18-28	M
**23**	29-38	M

Table S4. Participants' demographics and antiseizure medication. Antiseizure medication corresponds to the daily dose prescription.

Reference

Faivre, Nathan, Matthieu Roger, Michael Pereira, Vincent de Gardelle, Jean-Christophe Vergnaud, Christine Passerieux, et Paul Roux. « Confidence in Visual Motion Discrimination Is Preserved in Individuals with Schizophrenia ». *Journal of Psychiatry and Neuroscience* 46, n° 1 (février 2021): E65-73. https://doi.org/10.1503/jpn.200022.
